# RgsD negatively controls development, toxigenesis, stress response, and virulence in *Aspergillus fumigatus*

**DOI:** 10.1038/s41598-018-37124-2

**Published:** 2019-01-28

**Authors:** Young Kim, Min-Woo Lee, Sang-Cheol Jun, Yong-Ho Choi, Jae-Hyuk Yu, Kwang-Soo Shin

**Affiliations:** 1 Department of Life Science, Daejeon University, Daejeon, 34520 Republic of Korea; 2 Soonchunhyang Institute of Medi-bio Science, Soonchunhyang University, Chungcheongnam-do, 31151 Republic of Korea; 3 Departments of Bacteriology and Genetics, University of Wisconsin-Madison, Madison, WI 53706 USA

## Abstract

The regulator of G protein signaling (RGS) domain proteins generally attenuate heterotrimeric G protein signaling, thereby fine-tune the duration and strength of signal transduction. In this study, we characterize the functions of RgsD, one of the six RGS domain proteins present in the human pathogenic fungus *Aspergillus fumigatus*. The deletion (Δ) of *rgsD* results in enhanced asexual sporulation coupled with increased mRNA levels of key developmental activators. Moreover, Δ*rgsD* leads to increased spore tolerance to UV and oxidative stress, which might be associated with the enhanced expression of melanin biosynthetic genes and increased amount of melanin. Yeast two-hybrid assays reveal that RgsD can interact with the three Gα proteins GpaB, GanA, and GpaA, showing the highest interaction potential with GpaB. Importantly, the Δ*rgsD* mutant shows elevated expression of genes in the cAMP-dependent protein kinase A (PKA) pathway and PKA catalytic activity. The Δ*rgsD* mutant also display increased gliotoxin production and elevated virulence toward *Galleria mellonella* wax moth larvae. Transcriptomic analyses using RNA-seq reveal the expression changes associated with the diverse phenotypic outcomes caused by Δ*rgsD*. Collectively, we conclude that RgsD attenuates cAMP-PKA signaling pathway and negatively regulates asexual development, toxigenesis, melanin production, and virulence in *A. fumigatus*.

## Introduction

All living cells must adapt or respond to external and internal cues. In cells, environmental cues trigger intrinsic signaling cascades involving a variety of surface receptors and signal transduction elements. Heterotrimeric G proteins (G proteins) play a central role in these processes and G protein mediated signaling is mostly transmitted via cAMP dependent protein kinase A (PKA) pathway, mitogen activated protein kinase (MAPK) pathway, or protein kinase C (PKC) pathway^1–3^. A canonical G protein signaling pathway is activated by interaction of ligands and G protein-coupled receptors (GPCRs), which causes conformation changes in the GDP-bound Gα subunit leading to GDP to GTP exchange, and the subsequent dissociation of Gα -GTP and Gβγ subunit. Free Gα –GTP and Gβγ, or both can activate (or inhibit) a number of downstream signaling pathways. Regulator of G protein signaling (RGS) proteins enhance the intrinsic GTPase activity of Gα thereby accelerate the hydrolysis of GTP bound to Gα, leading the formation of the inactive Gα-GDP::βγ heterotrimer and turn off G protein signaling^3,4^.

Sst2 of *Saccharomyces cerevisiae* was the first identified RGS protein. Sst2 interacts with the Gα subunit Gpa1 and functions as a negative regulator of the pheromone response pathway^5,6^. The second RGS of *S*. *cerevisiae* Rgs2 attenuates the Gpa2-mediated signaling for glucose sensing through controlling the cAMP-dependent protein kinase (PKA) pathway^7^. While only two RGSs have been reported in *S*. *cerevisiae*, a plethora of RGSs exists in filamentous fungi^8^. RGSs of filamentous fungi regulate diverse signals that control vegetative growth, sporulation, stress responses, secondary metabolism, and virulence^9^. There are six RGS proteins (FlbA, GprK, RgsA, Rax1, RgsC, and RgsD) in the opportunistic human pathogen *Aspergillus fumigatus*. Among these, we have revealed that FlbA, GprK, and Rax1 play important roles in upstream regulation of G-protein and contribute to proper asexual development, secondary metabolites production, and stress responses^10,11^ Moreover, our recent study has revealed that RgsC is necessary for proper growth, sporulation, stress response, gliotoxin (GT) production, and external nutrients sensing in *A. fumigatus*^12^.

RgsD is known as a putative paralog of RgsA and its transcript is induced by growth under hypoxia condition (http://www.aspergillusgenome.org). RgsA of *Aspergillus nidulans* was shown to negatively regulate function of the Gα subunit GanB and regulate stress response and asexual sporulation^13^. As *A. fumigatus* and *A. nidulans* are distantly related, it is important to understand the exact functions of this key RGS protein, RgsD, in *A*. *fumigatus*. Our thorough characterization of the *rgsD* function has elucidated its broad repressive roles in asexual development, melanin production, stress response, GT biosynthesis, and virulence. Moreover, RgsD is required to properly control PKA activity. Collectively, understanding the target(s) of RgsD and downstream signaling cascades would provide a development of novel anti-fungal therapeutics against *A. fumigatus*.

## Results

### Summary of *A*. *fumigatus* RgsD

The ORF of *rgsD* of *A*. *fumigatus* Af293 (Afu5g00900) consists of 867 bp nucleotides with no intron, and is predicted to encode a 288 amino acid length protein. As shown in Supplementary Fig. S1A, the domain architecture of RgsD is very simple, containing just an RGS domain (14 to 147 aa, E-value; 5e-13) and two low complexity regions (218 to 230 aa and 278 to 286 aa). Based on the protein sequences, RgsD was aligned and compared with other putative RgsD-like proteins of *Aspergillus* (Supplementary Fig. S1B). While RgsD of *A*. *fumigatus* Af293 shares amino acid identity ranging from 34.1 to 82.3% with the RgsD-like proteins of *A*. *terreus* (ATET_09016), *A*. *clavatus* NRRL 1 (ACLA_088640), *A*. *oryzae* RIB40 (AO090023000043), and *Neosartorya fischeri* NRRL 181 (NFIA_041270), it shares low identity with the RgsA protein of *A*. *nidulans* (AN5755, 40.7%) and *A*. *niger* (An18g06110, 40.9%).

### RgsD negatively controls asexual development

**Figure 1:**
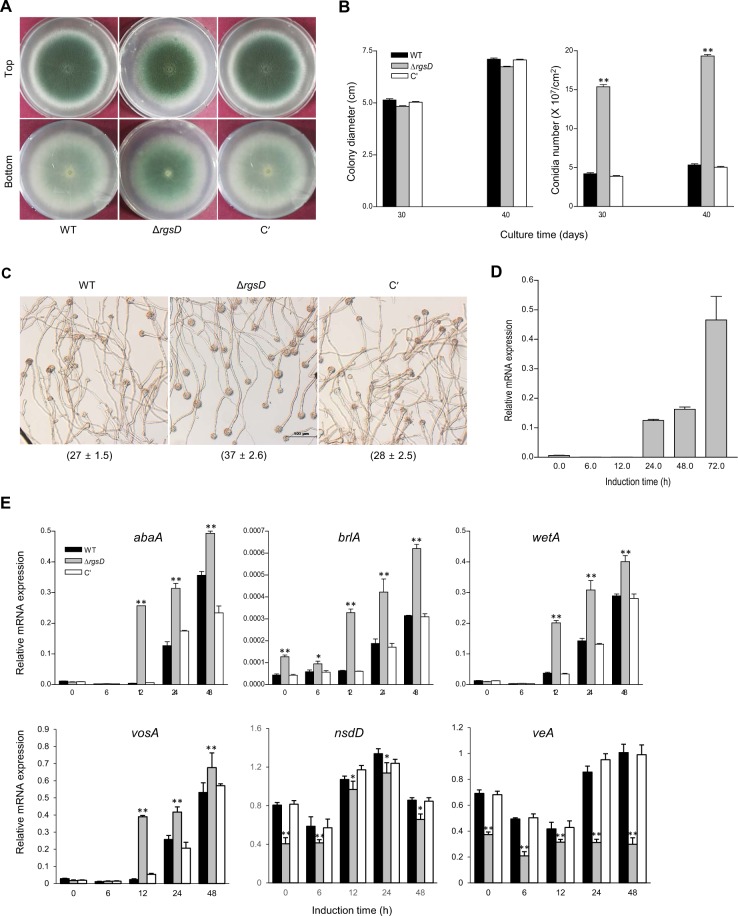
Negative regulation of asexual development by RgsD. (**A**) Colony photographs of WT (AF293), Δ*rgsD*, and complemented (C′) strains inoculated on solid MMY and grown for 3 days. The bottom panel shows the back side of plates. (**B**) Radial growth rates of three strains and conidia numbers produced by each strain per plate. Student’s *t*-test: *p < 0.05, **p < 0.01. (**C**) Photographs of WT, Δ*rgsD*, and C′ strains observed by liquid-slide culture. Number of total conidiophores per microscopic field were indicated in parenthesis. (**D**) Levels of *rgsD* mRNA during the life cycle of *A*. *fumigatus* WT. (**E**) mRNA levels of the asexual developmental regulators in three strains determined by quantitative PCR (qRT-PCR). Cultures were incubated in liquid MMY and mRNA levels were normalized using the *ef1α* gene, according to the ΔΔCt method. Data are expressed as the mean ± standard deviation from three independent experiments. Student’s *t*-test: *p < 0.05, **p < 0.01.

Previous studies have revealed that the RGS proteins FlbA, GprK, Rax1 (RgsB), and RgsC are positive regulators of asexual development as the conidia numbers were drastically decreased by the absence of any one of these RGS proteins^10–12,14^. To investigate functions of *rgsD*, we generated the *rgsD* null (Δ*rgsD*) mutant and complemented strains (C′). Although the Δ*rgsD* mutant demonstrated no significant change in radial growth, it formed very dark colonies with highly increased thallic density and significantly increased formation of mature conidiophores compared to wild type (WT) and C’ strains (Fig. 1A,C). Distinct from the other RGS mutants, the Δ*rgsD* mutant produced 4 fold more conidia than WT and C′ strains (Fig. 1B). Moreover, the Δ*rgsD* mutant produced a higher number of mature conidiophores (asexual developmental structures) than WT and C′ strains in liquid medium after 24 hours incubation (Fig. 1C). In WT and C’ strains, mRNA levels of *rgsD* were high at late stage of development whereas those of other asexual developmental regulators increase at earlier stage of development in WT (Fig. 1D). As shown in Fig. 1E, in the Δ*rgsD* mutant, mRNA levels of the key asexual developmental regulators *abaA*, *brlA*, *vosA*, and *wetA* were significantly increased at all time points tested. Conversely, mRNA levels of *nsdD* and *veA*, known negative regulators of *brlA* expression and conidiation, were low in the Δ*rgsD* mutant compared to WT and C′ (Fig. 1E). These results suggest that RgsD negatively controls conidiation and expression of developmental activators, but positively regulates nsdD and veA. Morevoer, as rgsD mRNA accumulates later phases of conidiation, it is likely that the RgsD protein (but not mRNA) is present and acting at early time points of life cycle and development, and the RgsD protein is re-generated during conidiogenesis.

### RgsD interacts with Gα protein in yeast

**Figure 2:**
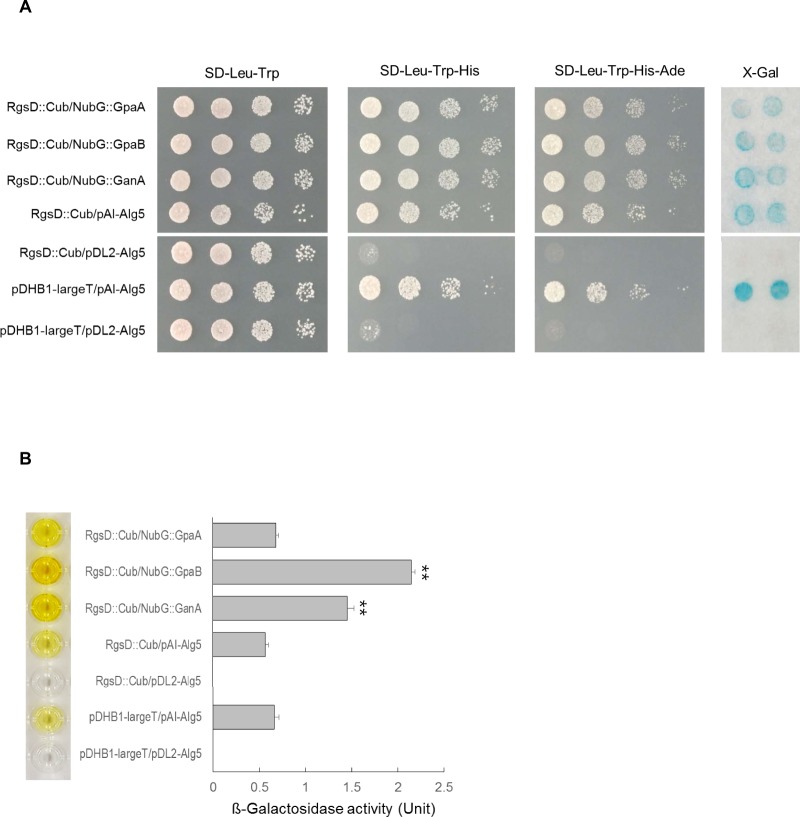
RgsD interacts with Gα proteins. (**A**) Interactions between RgsD and Gα proteins in the split-ubiquitin system. The C-terminal half of ubiquitin (Cub) was fused to the full-length RgsD cDNA. The N-terminal half of ubiquitin (NubG) was fused to the C-terminus of full-length GpaA (NubG::GpaA), GpaB (NubG::GpaA), and GanA (NubG::GanA). RgsD::Cub interaction with the control vector pAI-Alg5 served as a correct topology positive control, RgsD::Cub interaction with the empty vector pDL2-Alg5 served as a negative control. The pDHB1-largeT interaction with pAI-Alg5 served as a positive control for the assay. Yeast transformants were grown on the selective medium lacking histidine or adenine after serial dilution. (**B**) To verify the interaction, ß-galactosidase activity assays were performed. **p < 0.01.

As the RgsD protein contains a clear RGS domain, it is conceivable that RgsD might physically interact with one or more of the three Gα proteins (GpaA, GpaB, and GanA) present in *A. fumigatus*. To test this interaction potential, we carried out the split-ubiquitin yeast two-hybrid interaction assays with RgsD and three Gα proteins. RgsD::Cub plasmid was constructed by fusing Cub to the C-terminus of the full-length RgsD cDNA. Individual Gα protein constructs were generated by fusing full-length GpaA, GpaB, and GanA cDNA with the mutated N-terminal half of ubiquitin (NubG), respectively. As shown in Fig. 2A,B, those yeast transformants co-expressing RgsD::Cub/NubG::GpaB and RgsD::Cub/NubG::GanA grew better than positive control on the medium lacking histidine or adenine, and showed high levels of ß-galactosidase enzyme activity. Somewhat unexpectedly, those yeast transformants co-expressing RgsD::Cub/NubG::GpaA also grew on the medium lacking histidine or adenine, and showed ß-galactosidase enzyme activity equal to the positive control. These results indicate that RgsD may interact GpaB and GanA preferentially, but also has the ability to interact with GpaA.

### RgsD downregulates cAMP-dependent PKA signaling pathway

**Figure 3:**
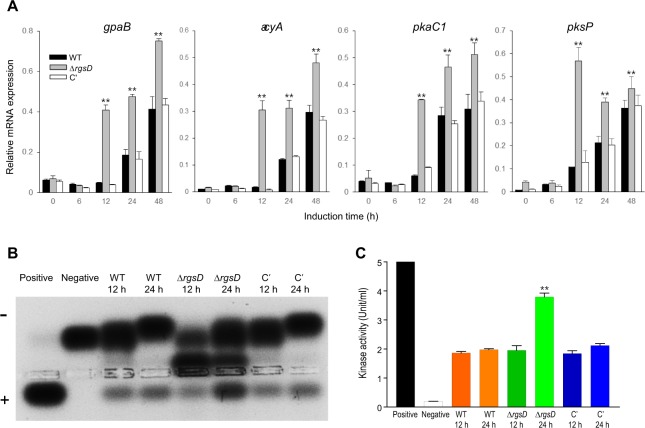
RgsD downregulates a cAMP-PKA signaling pathway. (**A**) Expression of *gpaB*, *acyA*, *pkaC1*, and *pksP* mRNA in three strains analyzed by qRT-PCR. Student’s *t*-test: **p < 0.01. (**B**) PKA activity of three strains as monitored by gel electrophoresis. A phosphorylated substrate migrates toward the anode (+). Each strain was grown in MMG for indicated time at 37 °C and mycelial extract was analyzed. (**C**) Kinase activity was determined by the spots (panel B) quantification using spectrophotometer. Note that mRNA levels of cAMP-PKA signaling pathway related genes and PKA activity at 24 hour were significantly increased in the Δ*rgsD* mutant compared to WT and C′ strains.

To investigate the role of RgsD in governing spore germination, we analyzed the kinetics of germ tube emergence in three strains. Although the germination of the Δ*rgsD* conidia was started slightly earlier, there were no significant differences between the mutant and WT (Supplementary Fig. S2). The components of the PKA signaling pathway in *A. fumigatus*, GpaB (Gα), AcyA (adenylyl cyclase), PkaC1 (the major catalytic subunit), and PksP (a polyketide synthase needed for spore pigmentation) are required for the proper growth, asexual sporulation, and conidial pigmentation^15–17^. Previous studies have demonstrated that the cAMP-PKA signaling pathway is attenuated by RgsA in *A*. *nidulans*^13,18,19^. To investigate the relationship between RgsD and cAMP-PKA signaling pathway, we analyzed mRNA levels of *gpaB*, *acyA*, *pkaC1*, and *pksP* mRNA. As shown in Fig. 3A, *gpaB*, *acyA*, *pkaC1*, and *pksP* mRNA levels were significantly higher in the Δ*rgsD* mutant than in WT and C′ strains. To investigate this further, we assessed PKA activity using a peptide substrate, kemptide, which is specifically recognized and phosphorylated by PKA. Proteins were extracted from each strain grown in MMG at indicated time and assayed for PKA activity. The phosphorylated negatively charged kemptide migrates to the anode and the signal is proportional to higher PKA activity in the protein extracts. While WT and C′ strains exhibited very little PKA activity in all tested protein extracts, the Δ*rgsD* mutant showed 2-fold higher PKA activity at 24 h than other samples (Fig. 3B,C). These results indicate that RgsD is required for proper control of expression of *gpaB*, *acyA*, *pkaC1*, and *pksP*, and may negatively regulate a cAMP-PKA signaling pathway.

### Repressive role of RgsD in stress response and melanin biosynthesis

**Figure 4:**
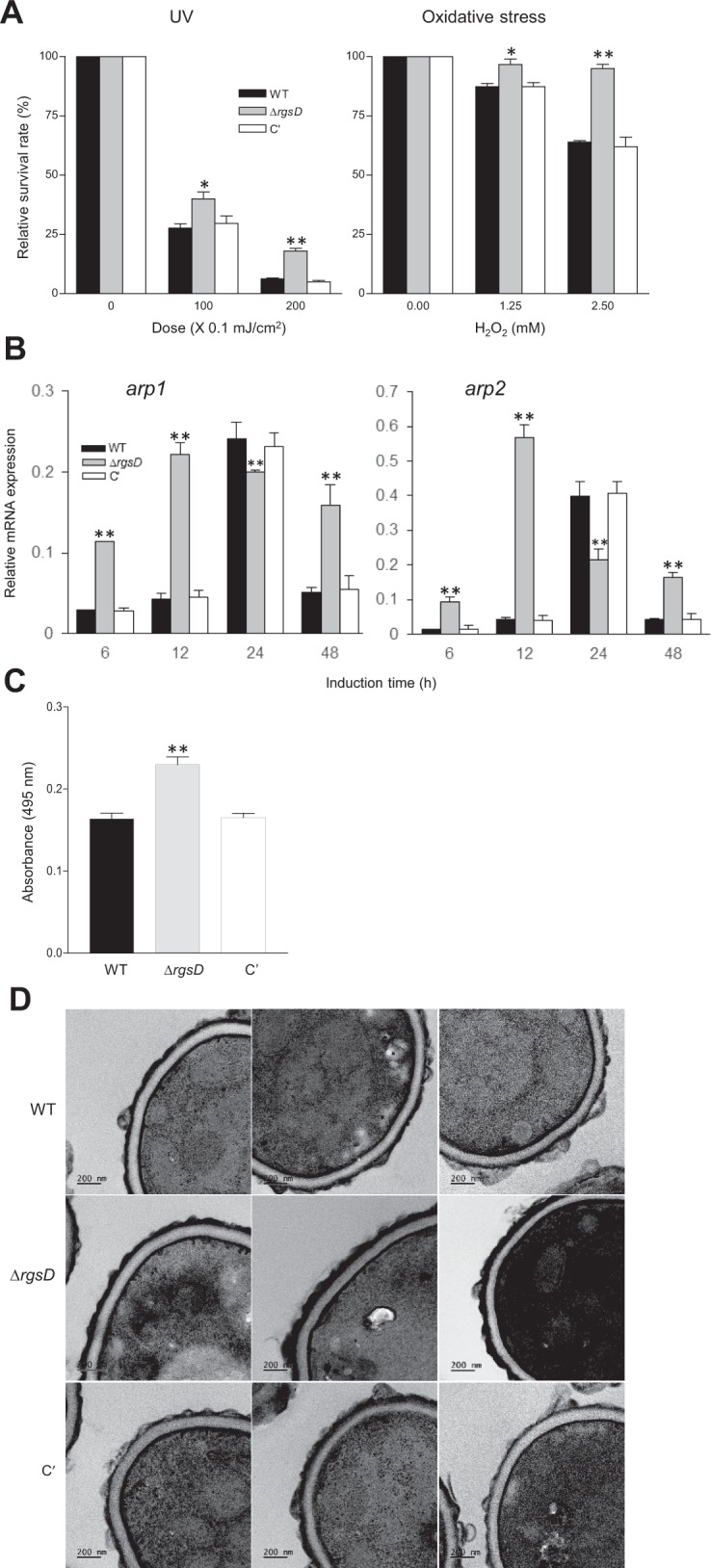
Inhibition of stress responses and melanin synthesis mediated by RgsD. (**A**) Conidial viability of WT, Δ*rgsD*, and C′ strains in presence of UV irradiation and H_2_O_2_ at indicated conditions. Note that the viability of the Δ*rgsD* conidia was significantly increased compared to WT and C′ strain’s conidia. (**B**) Expression of melanin biosynthetic genes *arp1* and *arp 2* mRNA analyzed by qRT-PCR. Student’s *t*-test: **p < 0.01. (**C**) Melanin content in three strains’ conidia. Statistical significance was determined by a Student’s *t*-test: **p < 0.01. (**D**) TEM photographs of WT, Δ*rgsD*, and C′ conidia. Note that the Δ*rgsD* conidia exhibited higher expression of melanin synthesis-related genes’ mRNA, melanin content, and the melanized outmost cell wall than WT and C′ strain.

To test a potential role of RgsD in stress response, the Δ*rgsD* conidia were exposed to UV and H_2_O_2_, and the survival rates were determined. As shown in Fig. 4A, the Δ*rgsD* conidia were more resistant than those of WT and C′ strains against UV irradiation. While the relative survival rates of conidia of WT and C′ strains were about 20%, survival rate of the Δ*rgsD* conidia was about 40% at 10 mJ/cm^2^. The difference was clearer at higher dose (20 mJ/cm^2^), where approximately 18% of the Δ*rgsD* conidia survived but only about 5% of conidia of WT and C′ strains remained alive (Fig. 4A). As shown in Fig. 4B, the conidia of the Δ*rgsD* mutant were also more resistant to H_2_O_2_. The survival rate of WT, mutant, and C′ conidia was about 65, 95, and 64%, respectively, at 2.5 mM H_2_O_2_. Noticeably, the mutant strain formed a strongly pigmented colony. As the proposed functions of melanin are protection against UV irradiation and scavenging reactive oxygen species, we examined mRNA levels of melanin biosynthetic genes, melanin content, and ultrastructure of conidia. The mRNA levels of *arp1* and *arp2* which function in dihydroxynaphthalene (DHN)-melanin pathway of fungi^20^ were maximum at 12 hours and gradually decreased in the mutant, whereas those were highest at 24 hours and rapidly decreased in WT and C′ strain (Fig. 4B). Moreover, the amount of melanin in the Δ*rgsD* conidia was about 1.5-fold higher than that of WT and C′ strains conidia (Fig. 4C). We next examined the cell wall architecture of the WT, Δ*rgsD*, and C′ conidia with transmission electron microscopy (TEM). The outermost melanized electron dense layer of *A*. *fumigatus* determines the conidial color and defends against external stresses^21^. The Δ*rgsD* conidia form a darker electron dense layer than those of WT and C′ (Fig. 4D). These indicate RgsD negatively controls stress response and melanin biosynthesis.

### RgsD negatively controls gliotoxin production

**Figure 5:**
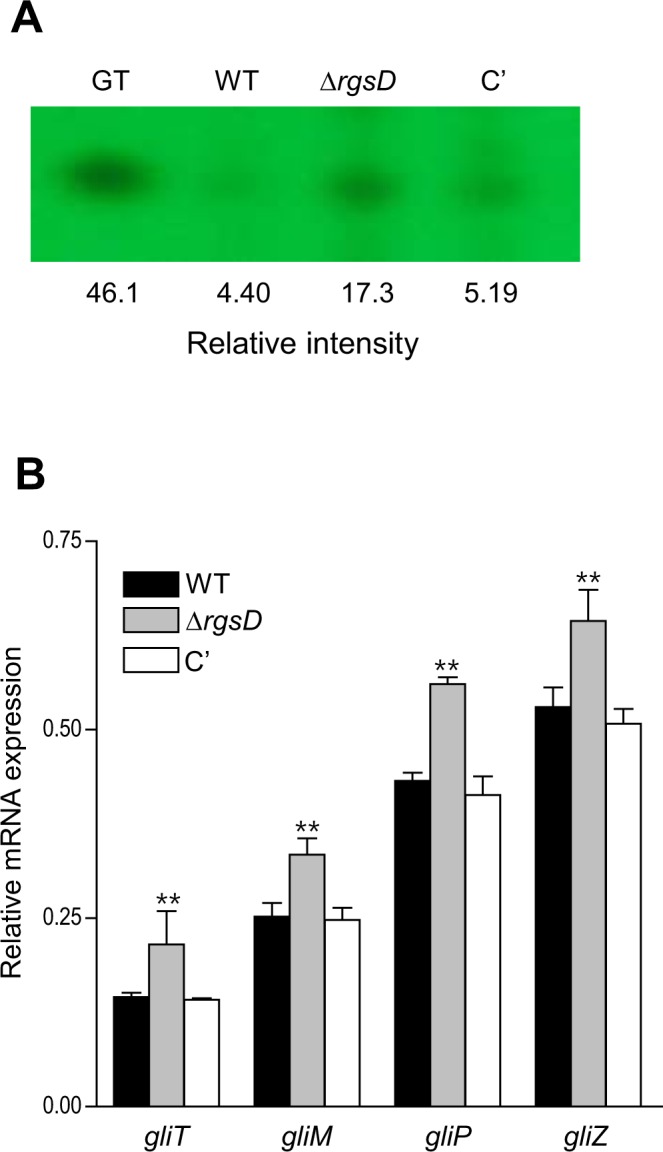
The negative role of RgsD in GT production. (**A**) Determination of GT production in WT, mutant, and C′ strains. The culture supernatant of each strain was extracted with chloroform and subjected to TLC. (**B**) qRT-PCR analysis of four GT synthetic genes in WT, Δ*rgsD*, and C′ strains. Statistical differences between WT and mutant strains were evaluated with Student’s unpaired *t*-test. **p < 0.01.

We showed that the key asexual developmental activator BrlA positively regulates gliotoxin (GT) production in *A*. *fumigatus*^22^. Moreover, we also showed that the deletion of RGSs including FlbA, GprK, Rax1, and RgsC resulted in lowered *brlA* mRNA levels and decreased GT production^10–12^. As the deletion of *rgsD* resulted in up-regulated *brlA* expression different from previously reported RGSs, we examined the effect of *rgsD* deletion in GT production. We assessed levels of GT in WT, Δ*rgsD*, and C’ strains, and found that the Δ*rgsD* mutant produced about 5-fold more GT (Fig. 5A). We then checked mRNA levels of key GT biosynthetic genes by qRT-PCR, and found that mRNA levels of *gliM*, *gliP*, *gliT*, and *gliZ* were significantly (*p* < 0.01) higher in the Δ*rgsD* mutant than in WT and C′ strains (Fig. 5B).

### Nullifying *rgsD* leads to enhanced virulence

**Figure 6:**
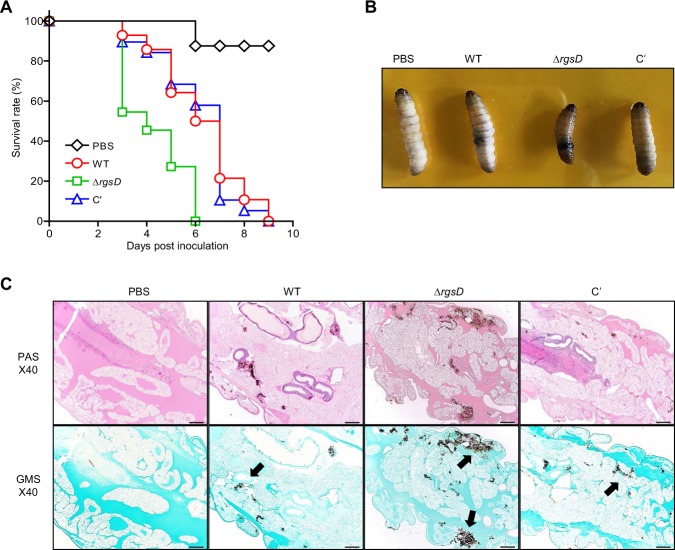
The lack of RgsD leads to enhanced virulence. (**A**) Log-Rank plots of the survival of *G*. *mellonella* after infection with three strains’ conidia. (**B**) Infection of *G*. *mellonella* with the Δ*rgsD* strain induced melanization of the larva. (**C**) Histological analysis of larval tissues infected by WT, mutant and C′ strains was performed using PAS and GMS staining 24 hours post inoculation. Arrows indicated fungal hyphae in infected larval tissues. Note that compared to the WT and C′ strain, Δ*rgsD* strain showed high virulence.

We also tested the effects of RgsD on virulence using the wax moth larvae *Galleria mellonella*. Conidia (1 × 10^6^) of WT, Δ*rgsD*, and C′ strains were inoculated to the larvae, and the survival rate of *G. mellonella* was recorded daily. The most rapid lethality occurred with the Δ*rgsD* mutant and this strain was significantly more virulent than the other challenge doses (*p* < 0.0001). As shown in Fig. 6A, larvae inoculated with conidia of either WT or C′ strains began to die at day 3 post-inoculation, with the number of survivors continuing to decrease over the course of the experiment. In the 7 days after infection, about 50% of larvae in both strains died. However, the mortality level of Δ*rgsD* strain was drastically higher than those of WT and C′ strains, all larvae tested died at 6 days after infection. We also observed that larvae infected with the mutant conidia darkened and shrank at 24 hours post inoculation. In contrast, larvae infected with WT and C′ strains remain in color and did not shrink at same time (Fig. 6B). Typically, infected *G*. *mellonella* larvae melanized by forming melanotic capsules surrounding pathogens^23,24^, and the observed darkening of the infected larvae may be due to the formation of these melanotic capsules. To better understand the fate of *A*. *fumigatus* inoculated into *G*. *mellonella*, infected larvae were fixed in neutral buffered formalin at 24 hours post infection and sectioned for histopathology. Figure 6C showed Periodic acid-Schiff (PAS) and Grocott methenamine-silver (GMS)-stained sections of uninfected (PBS) and infected larvae. The more fungal cells were observed in Δ*rgsD* conidia-infected larvae than those of WT and C′ conidia-infected larvae. No fungal cells were detected in the PBS-injected control larvae.

### Transcriptome analysis

**Figure 7:**
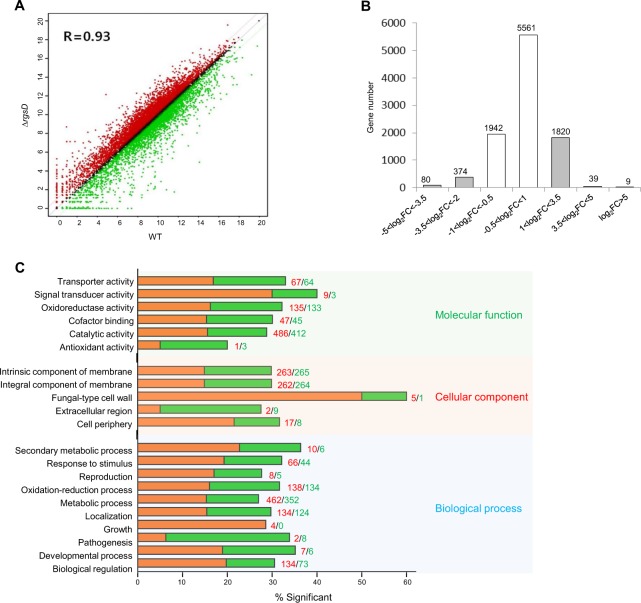
Genome-wide expression analyses of the Δ*rgsD* mutant and WT. (**A**) Linear fitted model showing the correlation between overall gene expression for WT and Δ*gprK* strains. The correlation coefficient R is indicated. (**B**) Histograms showing general transcriptomic results and columns in whites fall in the −1 < log_2_FC < 1 fragment count range with low differential expression values. (**C**) Functional categories of DEGs. The orange bars represent genes whose mRNA levels increased in the Δ*rgsD* strain, whereas the green bars represent those genes whose mRNA levels decreased in the Δ*rgsD* strain.

To investigate the roles of RgsD in *A*. *fumigatus* biology, we carried out RNA-sequencing analysis using the Δ*rgsD* and WT cells collected at 12 hours post asexual-developmental induction as described in the Methods section. Two biological replicates showed a high level of correlation (R = 0.93, Fig. 7A). 9,825 genes were differentially expressed and 7,503 genes expressed with slight fold change (−1 < log_2_FC < 1) (Fig. 7B). A total of 385 genes showed more than two-fold differential expression (*p* < 0.05), of which 168 genes exhibited higher transcript levels in Δ*rgsD* strain than in WT strain and 217 genes were down-regulated. Functional category analysis was carried out by determining Gene Ontology (GO) terms that were enriched in differentially expressed genes (DEGs). The top significant molecular function GO categories are “signal transducer activity”, “transporter activity”, “oxidoreductase activity”, “cofactor binding activity”, and “catalytic activity”. The top significant cellular component GO categories are “fungal-type cell wall”, “cell periphery”, “intrinsic and integral component of membrane”, and “extracellular region”. The top significant biological process GO categories are “secondary metabolic process”, “developmental process”, and “pathogenesis”. The most enriched molecular function, cellular component, and biological process GO categories are “catalytic activity”, “intrinsic component of membrane”, and “metabolic process”, respectively (Fig. 7C). The top 20 DEGs with increased mRNA accumulation levels in Δ*rgsD* strain compared to WT are listed in Table 1. Notably, mRNA levels of the key regulator of asexual sporulation *brlA* and the melanin synthesis-related gene *abr1* were above 8-fold higher in the Δ*rgsD* mutant than WT, consistent with our phenotypic data. Numerous genes involved in signal transduction, asexual development, cAMP-dependent PKA signaling pathway, and pigment biosynthesis were also up-regulated in the absence of *rgsD* (Supplementary Fig. S3). Most of the down-regulated genes were those encoding hypothetical proteins (Table 2), including the MgtC/SapB family membrane protein which are homologous to a bacterial virulence factor but their function was still not known^25^.

**Table 1:** Top 20 up-regulated genes in Δ*rgsD* strain relative to WT strain (>4.0-fold, *p* < 0.01).

Gene Symbol	Gene Name	Protein Name	Log_2_FC	*p*-value	
				Normal	FDR
CADAFUAG00000130	AFUA_3G03080	endo-1,3(4)-beta-glucanase	4.629	0.003	0.000
CADAFUAG00007546	AFUA_1G17680	MFS transporter	4.369	0.000	0.019
CADAFUAG00000069	AFUA_3G02680	C6 finger domain protein	4.353	0.001	0.011
CADAFUAG00000150	AFUA_3G00620	zinc-containing alcohol dehydrogenase	4.05	0.000	0.000
CADAFUAG00002469	AFUA_4G01270	integral membrane protein	3.674	0.000	0.000
CADAFUAG00002370	AFUA_4G00510	hypothetical protein	3.653	0.000	0.000
CADAFUAG00000579	AFUA_6G01920	xenobiotic compound monooxygenase, DszA family	3.581	0.000	0.015
CADAFUAG00009057	AFUA_1G02750	hypothetical protein	3.534	0.001	0.000
CADAFUAG00009481	AFUA_7G08610	Ankyrin repeat protein	3.433	0.008	0.000
CADAFUAG00000294	AFUA_3G01370	MFS transporter	3.354	0.000	0.011
CADAFUAG00004595	AFUA_2G17920	conserved hypothetical protein	3.301	0.000	0.007
CADAFUAG00000372	AFUA_3G01360	siderochrome-iron transporter	3.277	0.000	0.000
CADAFUAG00000379	AFUA_3G01130	cell wall protein	3.274	0.000	0.005
CADAFUAG00002824	AFUA_5G02770	conserved hypothetical protein	3.235	0.000	0.000
CADAFUAG00006863	AFUA_1G16590	C2H2 type conidiation transcription factor BrlA	3.177	0.000	0.018
CADAFUAG00006460	AFUA_5G08180	cell wall protein	3.152	0.000	0.005
CADAFUAG00004821	AFUA_2G17540	conidial pigment biosynthesis oxidase Abr1/brown 1	3.112	0.000	0.006
CADAFUAG00008146	AFUA_4G09480	extracellular endo-1,4-beta-xylanase	3.078	0.000	0.000
CADAFUAG00001908	AFUA_6G12060	MAK1-like monooxygenase	3.075	0.000	0.013
CADAFUAG00006091	AFUA_5G06680	4-aminobutyrate transaminase GatA	3.064	0.000	0.020

**Table 2:** Top 20 down-regulated genes in Δ*rgsD* strain relative to WT strain (>4.0-fold, *p* < 0.01).

Gene Symbol	Gene Name	Protein Name	Log_2_FC	*p*-value	
				Normal	FDR
CADAFUAG00009615	AFUA_7G05050	hypothetical protein	−7.238	0.000	0.004
CADAFUAG00009586	AFUA_7G05490	conserved hypothetical protein	−6.323	0.000	0.004
CADAFUAG00009591	AFUA_7G04850	aldo-keto reductase	−5.825	0.000	0.025
CADAFUAG00002129	AFUA_6G09610	nonribosomal peptide synthase	−5.753	0.000	0.002
CADAFUAG00001516	AFUA_6G00260	phosphatidylserine decarboxylase family protein	−5.45	0.000	0.000
CADAFUAG00009416	AFUA_7G05060	MgtC/SapB family membrane protein	−5.267	0.000	0.005
CADAFUAG00008526	AFUA_4G09930	conserved hypothetical protein	−5.113	0.000	0.018
CADAFUAG00009661	AFUA_7G06270	cyanamide hydratase	−5.011	0.000	0.000
CADAFUAG00009491	AFUA_7G04840	hypothetical protein	−4.951	0.000	0.033
CADAFUAG00000328	AFUA_3G03570	67 kDa myosin-cross-reactive antigen family protein	−4.895	0.000	0.000
CADAFUAG00002126	AFUA_6G09745	conserved hypothetical protein	−4.607	0.000	0.022
CADAFUAG00003719	AFUA_2G06000	NAD ^+^ dependent glutamate dehydrogenase	−4.56	0.000	0.017
CADAFUAG00000008	AFUA_3G02060	MFS multidrug transporter	−4.444	0.000	0.000
CADAFUAG00001599	AFUA_6G11980	exo-beta-1,3-glucanase	−4.422	0.002	0.025
CADAFUAG00001455	AFUA_4G03250	hypothetical protein	−4.418	0.000	0.000
CADAFUAG00001244	AFUA_7G00960	extracellular cysteine-rich protein	−4.34	0.000	0.000
CADAFUAG00000998	AFUA_8G02280	C6 transcription factor	−4.284	0.000	0.008
CADAFUAG00007109	AFUA_1G11350	MFS transporter of unkown specificity	−4.275	0.000	0.042
CADAFUAG00002750	AFUA_5G00410	conserved hypothetical protein	−4.227	0.000	0.008
CADAFUAG00009533	AFUA_7G05190	MFS alpha-glucoside transporter	−4.072	0.003	0.000

## Discussion

RGS proteins are GTPase accelerating proteins (GAPs) that act as negative regulators of multifunctional signaling in many fungi^10–12,14,26,27^. They function by enhancing the intrinsic GTPase activity leading to accelerated hydrolysis of GTP-Gα subunits of heterotrimeric G protein and inactivation of the trimers^28–30^. Since RGS proteins are pathologically and physiologically important regulators of the signaling pathways, elucidating the regulatory mechanisms of RGS proteins will provide an expanded basis for understanding G protein signaling and for controlling human pathogenic fungi.

Among six potential RGS proteins identified in *A*. *fumigatus*, previous studies revealed that the RGS proteins FlbA, GprK, Rax1 (RgsB), and RgsC play redundant roles in regulating asexual sporulation, stress response, and virulence^10–12,14^. FlbA (Afu2g11180) regulates GpaA signaling and contributes to proper progression of asexual sporulation^10^. RgsB (Afu4g12640), the orthologue of *Saccharomyces cerevisiae* Rax1, also controls growth and asexual development of *A*. *fumigatus*. Vegetative growth and conidia number is highly reduced in the absence of *rgsB*. Moreover, it also modulates the content of trehalose and melanin in conidia, providing resistance against external oxidative stress^12^. Another RGS protein RgsC (Afu1g09040) is needed for proper growth, conidiation, stress response, and GT production. The deletion of *rgsC* results in impaired growth and asexual sporulation^14^. The GPCR-RGS hybrid protein GprK (Afu4g01350) down-regulates the PKA-mediated germination pathway and is responsible for normal asexual development. The Δ*gprK* mutant shows severely impaired asexual sporulation and reduced tolerance to oxidative stresses^11^.

Despite having a very simple domain structure (Supplementary Fig. S1A), RgsD may play an opposite role in regulating signaling compared to previously reported RGSs. The deletion of *rgsD* causes high production of conidia (about 4 fold) coupled with increased expression of key developmental regulators, but reduced the expression of *nsdD* and *veA* (Fig. 1). NsdD is known as a major negative regulator of *brlA* expression and the Δ*nsdD* mutant produced more conidia than WT in *A*. *fumigatus*^31^. VeA also acts as a negative regulator in conidiation. The absence of *veA* results in highly increased production of conidiophores and accumulation of *brlA* mRNA^32^. These findings indicate that RgsD may function as a negative regulator of conidiation and some developmental regulatory genes.

With the split-ubiquitin system, it appears that RgsD can interact the three Gα proteins in *A. fumigatus*. However, the data clearly indicate that RgsD might preferentially interact with GpaB, then GanA and GpaA (Fig. 2). Previous studies showed that GpaB stimulates cAMP-PKA signaling^15^. We demonstrated that the *rgsD* mutant showed the higher mRNA levels of PKA signaling components and PKA activity, which implicates that RgsD serves as an important negative regulator of cAMP-PKA signaling (Fig. 3). In *A*. *fumigatus*, the cAMP-PKA signaling cascade composed G protein α subunit GpaB, adenylate cyclase AcyA, and protein kinase A, regulates growth, development, and secondary metabolites synthesis. Deletion of PKA catalytic subunit *pkaC1* showed reduced conidiation, growth, and pigment formation^15^. PKA activity was not detected in Δ*gpaB* and Δ*acyA* strains and conidia of both strains were almost avirulent^33^. Moreover, elevated PKA activity led to high expression of polyketide synthase gene *pkaP* which is essential for the production of melanin and pathogenicity^15^. As expected, the Δ*rgsD* mutant was more resistant to UV irradiation and oxidative stress, and mRNA of *arp1* and *arp2* which encode scytalone dehydratase and hydroxynaphthalene reductase, respectively, accumulated more rapidly and at higher levels in the Δ*rgsD* mutant. Furthermore, the content of melanin in the Δ*rgsD* conidia was higher and the mutant conidia formed thicker electron dense melanized layer than those of WT and C′ strain (Fig. 4), indicating that RgsD downregulates cAMP-PKA signaling pathway and plays an important negative role in melanin biosynthesis that may modulate fungal response to external stresses.

Synthesis and regulation of GT is done by the *gli* cluster composed of 13 genes in *A*. *fumigatus*^34,35^. We checked the GT content and mRNA levels of four genes, *gliM* (*o*-methyltransferase), *gliP* (dioxopiperazine synthase), *gliT* (GT oxidoreductase), and *gliZ* (zinc finger transcriptional regulator) in three strains. As shown in Fig. 5, both GT production and GT biosynthetic genes mRNA levels increased in the absence of *rgsD*, suggesting that RgsD serves as a negative regulator of GT synthesis.

It is well-known that both melanin and GT are important virulence determinants of *A*. *fumigatus*. DHN-melanin specifically interferes the functions of host phagocytes, leading to survival of the fungus within host cell^36^. GT inhibits the oxidative burst of neutrophil and increases the ability of the fungus to survive against neutrophil’s attack^37^. As Δ*rgsD* strain produces higher amount of melanin and GT, it was conceivable that the mutant strain would be more virulent than WT and C′ strain. To confirm this, we performed virulence studies with insect wax moth larvae. The Δ*rgsD* conidia were highly virulent to the insect and killed all the larvae within 6 days post inoculation. We readily observed that larvae infected with the mutant conidia darkened at 24 hours inoculation and it seemed to be a positive correlation between the degree of larvae darkening and the level of pathogenicity of the strain. Furthermore, higher density of fungal cell was found in the Δ*rgsD* mutant infected larvae gut (Fig. 6). From these results we conclude that RgsD modulates (attenuates) virulence likely by downregulating biosynthesis of melanin, GT, and potentially other virulence factors in *A*. *fumigatus*.

**Figure 8:**
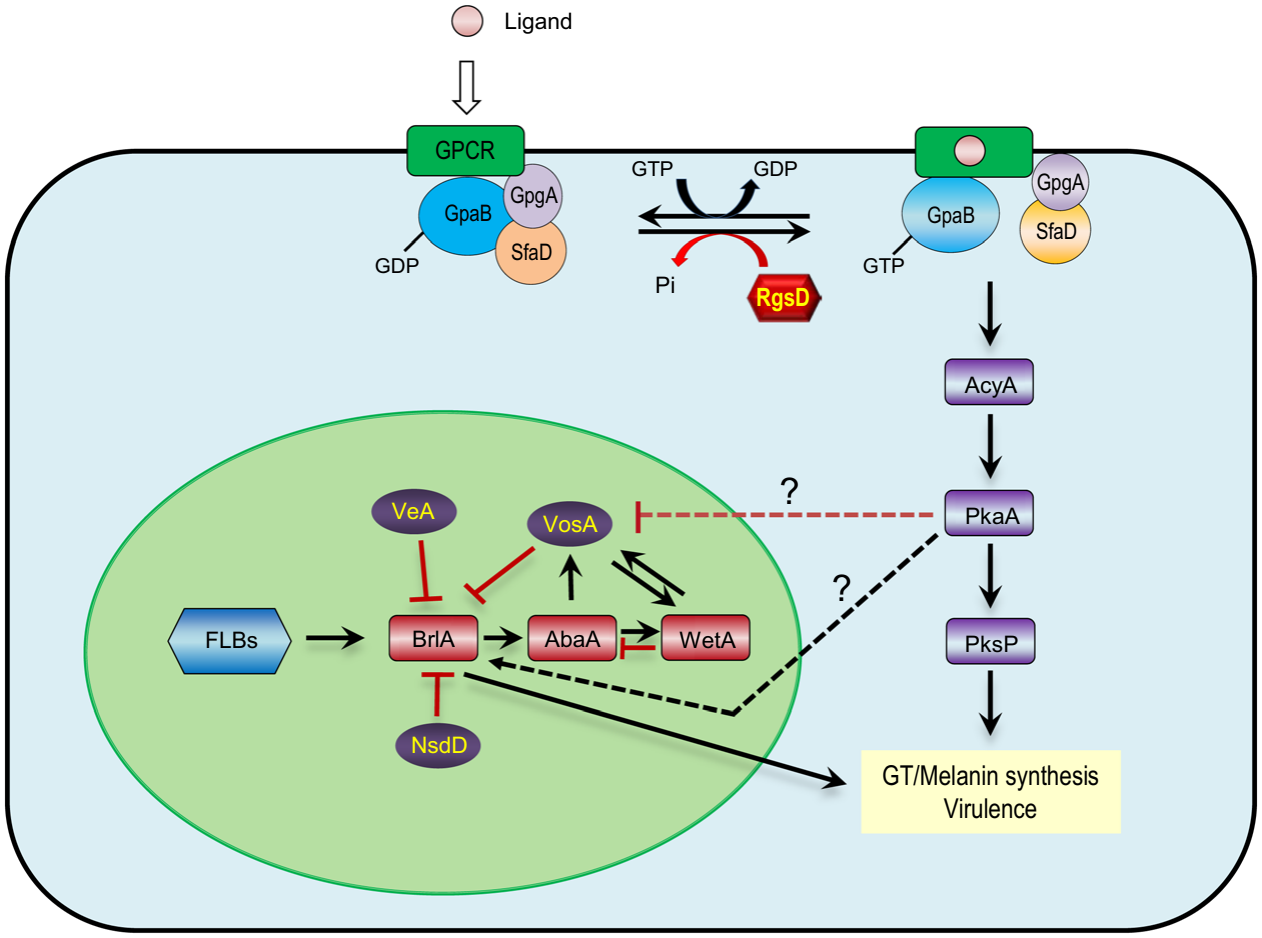
A model depicting the roles of RgsD. The putative RGS domain protein RgsD negatively regulates the cAMP-PKA signaling pathway, which may be activated by the Gα subunit GpaB. Consequence of the lack of RgsD leads to enhanced activation of the cAMP-PKA signaling pathway, which in turn leads to enhanced production of GT and melanin, and hyper-virulence of the fungus. The cAMP-PKA pathway may remove the repressive effects of VosA/VeA/NsdD on *brlA* and/or may directly activate *brlA* expression.

Comparative transcriptomics of WT and the Δ*rgsD* mutant was used for the investigation of RgsD regulated target genes and signaling pathways. Notably, the C_2_H_2_ type transcription factor BrlA and the conidial pigment biosynthesis oxidase Abr1 were significantly induced in mutant strain (Table 1). BrlA regulates diverse biological processes in *A*. *fumigatus* including asexual sporulation and GT production^22^. Abr1 encodes a multicopper oxidase that plays an important role in the biosynthesis of DHN-melanin^20^. Biosynthesis of DHN-melanin in *A*. *fumigatus* begins with the conversion of acetyl-CoA and malonyl-CoA to 1,3,6,8-tetrahydroxynaphthalene (1,3,6,8-THN) by PksP (polyketide synthase) and Ayg1 (heptaketide hydrolyase). 1,3,6,8-THN is reduced to scytalone by the hydroxynaphthalene (HN) reductase Arp2, followed by dehydration of scytalone to 1,3,8-trihydroxynaphthalene (1,3,8-THN) by Arp1 (scytalone dehydratase). Then, Arp1 and Arp2 catalyze the reduction and dehydration of 1,3,8-THN to 1,8-dihydroxynaphthalene (1,8-DHN). Oxidative polymerization of 1,8-DHN to form DHN-melanin catalyzed by multicopper oxidase Abr1 and laccase Abr2^20,38,39^. The results of the RNA-seq analysis demonstrate the diversity in cellular processes especially, asexual sporulation and DHN-melanin synthesis regulated by RgsD in *A*. *fumigatus*. Collectively, we propose a genetic model depicting the regulatory roles of RgsD in *A*. *fumigatus* (Fig. 8). In the absence of RgsD, the cAMP-PKA signal transduction pathway may be consistently activated, which might lead to increased melanin and secondary metabolite production, tolerance to environmental stresses, and virulence.

## Methods

### Strains and culture conditions

Glucose minimal medium (MMG) and MMG with 0.1% yeast extract (MMY) with appropriate supplements were used for general culture of *A*. *fumigatus* strains^40^. For liquid submerged culture and phenotypic analyses on air-exposed culture were performed as described previously^11^. To examine secondary metabolite production, spores of relevant strains were inoculated 50 ml of liquid MMY and incubated at 250 rpm at 37 °C for 4 days.

### Generation of the *rgsD* deletion mutant

The oligonucleotides used in this study are listed in Supplementary Table S1. The *rgsD* gene was deleted in *A*. *fumigatus* AF293.1 (*pyrG1*) strain^41^. The deletion construct generated employing double-joint PCR (DJ-PCR)^42^ containing the *A*. *nidulans* selective marker (*AnpyrG*) with the 5′ and 3′ flanking regions of the *rgsD* gene was introduced into the recipient strain AF293.1^43^. The selective marker was amplified from *A*. *nidulans* FGSC4 genomic DNA with the primer pair oligo 109/oligo 110. The Δ*rgsD* mutant was isolated and confirmed by PCR, followed by restriction enzyme digestion (Supplementary Fig. S4)^42^. To complement *rgsD* null mutant, a single joint PCR (SJ-PCR) method was used^42^. The ORF of *rgsD* gene with a promoter and terminator was amplified with primer pairs where the 3′ reverse primer carries overlapping sequences with the *hygB* gene’s 5′ end. Amplification of the *hygB* gene was carried out with primer pairs where the 5′ forward primer carries overlapping sequences with *hygB* gene’s 3′ end. The final amplicon was amplified with the nested primer pair oligo 562/oligo 563 and introduced into a Δ*rgsD* strain.

### Nucleic acid isolation and manipulation

Total RNA isolation and quantitative RT-PCR (qRT-PCR) assays were performed as previously described^10,22,44^. Expression of target genes mRNA was analyzed with appropriate oligonucleotide pairs (Table S1). For RNA-seq analyses, 12 hours-old culture of WT and mutant strains were harvested from solid MMY. Total RNA was extracted and submitted to eBiogen Inc. (Seoul, Korea) for library preparation and sequencing.

### Phenotypic analyses

To examine germination levels, conidia of WT and mutant were inoculated in 5 ml of liquid MMY and incubated at 37 °C. Levels of germination were examined every 2 hours after inoculation under a microscope. Conidia were collected in 0.5% Tween 80 from the entire colony and filtered through Miracloth (Calbiochem, CA), and counted using a hemacytometer. Hydrogen peroxide sensitivity of conidia was examined by incubating 1 ml of spore suspensions containing 10^5^ conidia with varying concentrations (0, 1.25 or 2.50 mM) of H_2_O_2_ and incubated for 30 min at room temperature. Each spore suspension was diluted with sterilized H_2_O, and conidia were inoculated into solid MMY. After incubation at 37 °C for 48 hours, colony numbers were counted and calculated as a survival ratio of the untreated control. UV tolerance test was carried out as follows. Fresh conidia were plated out on solid MMY plates (100 conidia per plate). The plates were irradiated immediately with UV using a UV cross-linker at indicated doses and incubated at 37 °C for 48 hours. The colony numbers were counted and calculated as a survival ratio of the untreated control. The production of gliotoxin (GT) was determined as described previously^45^. The TLC silica plate (Kiesel gel 60, E. Merck) was developed with toluene:ethyl acetate:formic acid (5:4:1, v/v/v). Melanin content was determined from conidia as described^46^. Briefly, the conidia were harvested and washed with H_2_O, then incubated in 1.0 M NaOH at 98 °C and pelleted for 10 min at 17,000 g. Melanin in the supernatants was precipitated for 30 min at pH 1.0 by addition of 12 N HCl, after which it was pelleted at 17,000 g. After washing with H_2_O, samples dissolved in 0.05 M NaOH and the pH of the melanin solution was set at 7.5 using 1.0 M HCl. Absorbance at 495 nm was used to quantify melanin in biological duplicates.

### Protein-protein interaction assay

Yeast two-hybrid interaction assays were performed using DUALhunter Kit (Dualsystem Biotech, Switzerland) according to the manufacturer’s instruction. Briefly, full-length RgsD cDNA was cloned into yeast expression vector pDHB1 and cDNAs of GpaA, GpaB, and GanA were cloned into the pPR3-N vector. All cDNA sequences were confirmed by DNA sequencing. Cub and NubG fusion constructs were co-introduced into host yeast strain NMY51. pAI-Alg5 contains a wild type Nub serves as a positive control and pDL2-Alg5 serves as a negative control. Interaction was determined by the growth of yeast transformants on medium without histidine or adenine, and by measuring the β-galactosidase activity using Yeast ß-Galactosidase Assay Kit (Thermo Scientific, USA).

### PKA assay

For determination of PKA activity, Pep Tag Non-Radioactive cAMP-Dependent Protein Kinase Assay kit (Promega, USA) was applied according to the manufacturer’s instructions. Homogenized mycelia of each strain were suspended in extraction buffer^33^ and incubated on ice for 15 min. After centrifugation, 10 µl of supernatant adjusted to a protein concentration of 3 mg/ml was used in an assay for PKA activity.

### TEM

Conidia were fixed in 2.5% glutaraldehyde in 0.1 M phosphate, washed three times with 0.1 M phosphate, post-fixed in 1% osmium tetroxide, incubated for 1 hour in 0.1 M phosphate and dehydrated for 15 min in a graded methanol series from 50% to 100%. Samples were embedded in Epon resin 812. The sections were examined with a Tecnai G2 Spirit Twin Bio-Transmission Electron Microscope (FEI, Hillsboro, USA), with an accelerating voltage of 120 KV.

### Insect infection model

The insect survival assay was performed as previously described with some modifications^47^. Briefly, sixth instar *Galleria mellonella* were infected by injecting the fresh conidia (1 × 10^5^) into the last left pro-leg and incubated at 37 °C in the dark for the duration of the experiment. Larvae were checked daily for survival and Kaplan-Meier survival curves were analyzed using the Log-Rank (Mantel-Cox) test for significance (*p* < 0.0001).

### RNA-seq experiment

For control and test RNAs, the construction of library was performed using SENSE mRNA-Seq Library Prep Kit (Lexogen, Inc., Austria) according to the manufacturer’s instructions. Briefly, each 2 µg total RNA are prepared and incubated with magnetic beads decorated with oligo-dT and then other RNAs except mRNA was removed. Library production is initiated by the random hybridization of starter/stopper heterodimers to the poly(A) RNA still bound to the magnetic beads. These starter/stopper heterodimers contain Illumina-compatible linker sequences. A single-tube reverse transcription and ligation reaction extends the starter to the next hybridized heterodimer, where the newly-synthesized cDNA insert is ligated to the stopper. Second strand synthesis is performed to release the library from the beads, and the library is then amplified. Barcodes were introduced when the library is amplified. High-throughput sequencing was performed as paired-end 100 sequencing using HiSeq. 2000 (Illumina, Inc., USA). RNA-Seq reads were mapped using TopHat software tool in order to obtain the alignment file. The alignment file was used for assembling transcripts, estimating their abundances and detecting differential expression of genes or isoforms using cufflinks. Gene classification was based on searches done by BioCarta (http://www.biocarta.com/), GenMAPP (http://www.genmapp.org/), DAVID (http://david.abcc.ncifcrf.gov/), and Medline databases (http://www.ncbi.nlm.nih.gov/).

## Supplementary information

Supplementary Informaton

## Data Availability

The RNA-seq data are available from NCBI Gene Expression Omnibus (GEO) database (the accession number is GSE100101).
