# Supplementary material for: RgsD negatively controls development, toxigenesis, stress response, and virulence in Aspergillus fumigatus
Source: Sci Rep. 2019 Jan 28;9:811. doi: 10.1038/s41598-018-37124-2 (PMC6349852; doi:10.1038/s41598-018-37124-2)
Supplement: Supplementary file 1 — Supplementary Informaton [file 41598_2018_37124_MOESM1_ESM.pdf]

Supplementary Information

RgsD negatively controls development, toxigenesis, stress response, and virulence in *Aspergillus fumigatus*

Young Kim [1], Min-Woo Lee [2], Sang-Cheol Jun[1], Yong-Ho Choi [1], Jae-Hyuk Yu[3*] & Kwang-Soo Shin[1*]

[1]Department of Life Science, Daejeon University, Daejeon, 34520, Republic of Korea. [2]Soonchunhyang Institute of Medi-bio Science, Soonchunhyang University, Chungcheongnam-do, 31151, Republic of Korea. [3]Departments of Bacteriology and Genetics, University of Wisconsin-Madison, Madison, WI 53706, USA.

Young Kim and Min-Woo Lee contributed equally to this work.

*Correspondence and requests for materials should be addressed to J.H.Y. (email: jyu1@wisc.edu) or K.S.S. (email: shinks@dju.kr)

Running title: Roles of *Aspergillus fumigatus* RgsD

Figure S1. Synopsis of RgsD in *A. fumigatus*. (A) Predicted *A. fumigatus* RgsD and other *Aspergillus* RgsD-like proteins are presented schematically using SMART (http://smart.embl-heidelberg.de). (B) Multiple sequence alignment of *Aspergillus* RgsD-like proteins using MUSCLE (https://www.ebi.ac.uk/Tools/msa/muscle/). ATET_09016: *A. terreus* NIH2624, NFIA_041270: *Neosartorya fischeri* NRRL 181, AO090023000043: *A. oryzae* RIB40 RgsD, AN5755: *A. nidulans* FGSC A4 RgsA, ACLA_088640: *A. clavatus* NRRL 1, An18g06110: *A. niger* CBS 513.88 RgsA.

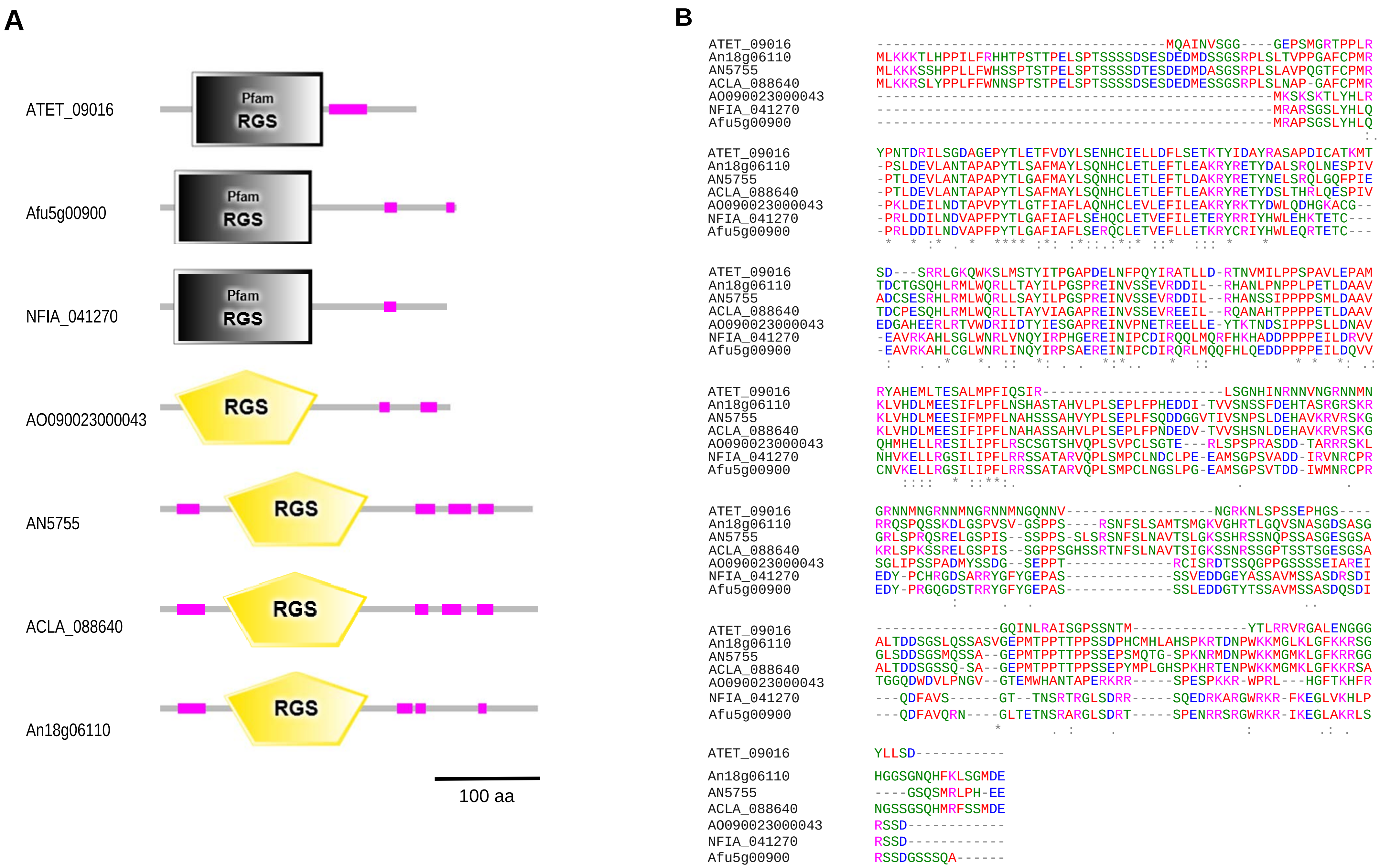

Figure S2. Germination of conidia. (A) Kinetics of germ outgrowth in *A. fumigatus* strains when inoculated in liquid MMG at 37°C. (B) Photographs of germinated conidia at 6 and 10 hours after inoculation.

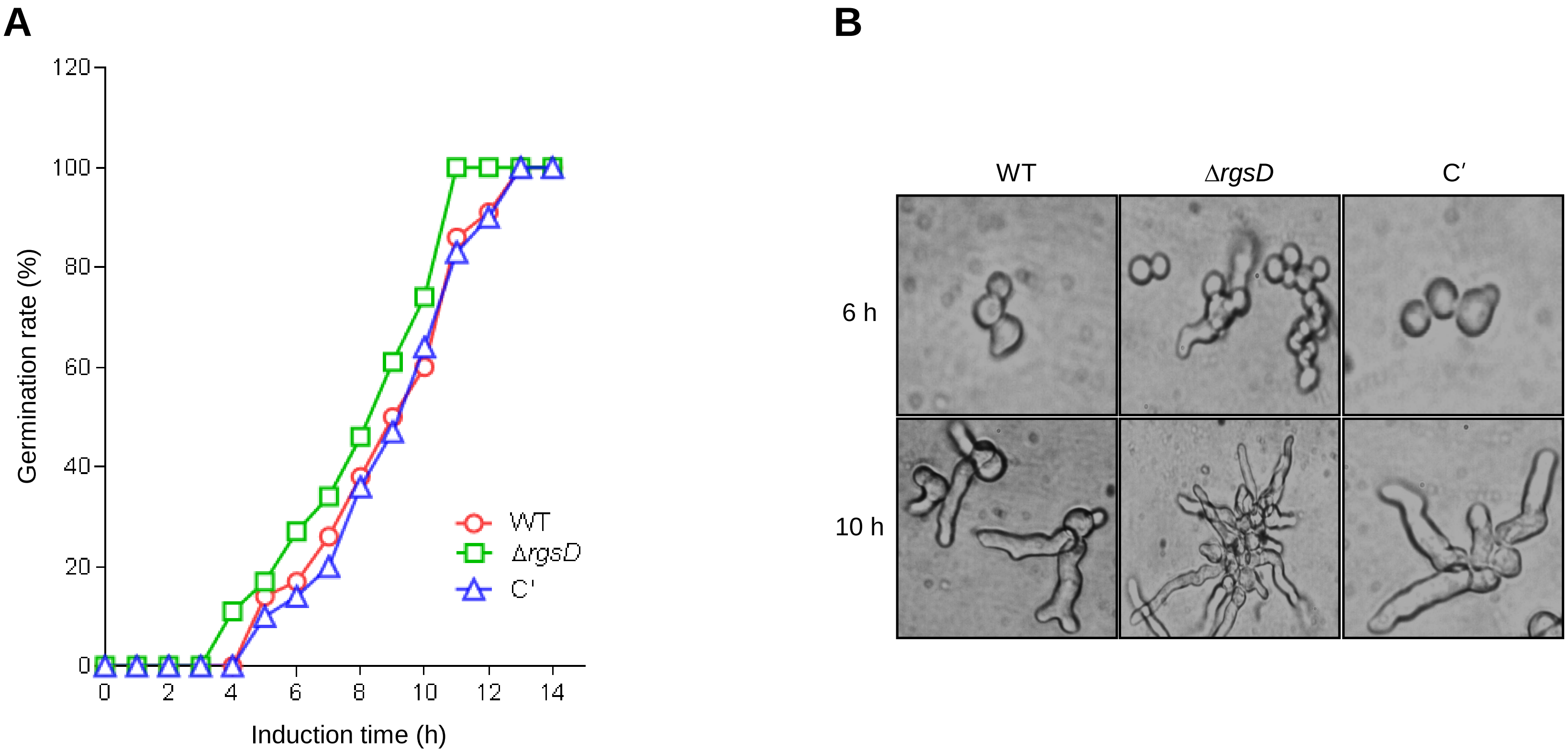

Figure S3. Heat map illustration of expression level changes between WT and Δ*rgsD* strains. Asexual development genes, cAMP-dependent PKA signaling genes, and pigment synthesis genes are up-regulated in the absence of *rgsD*.

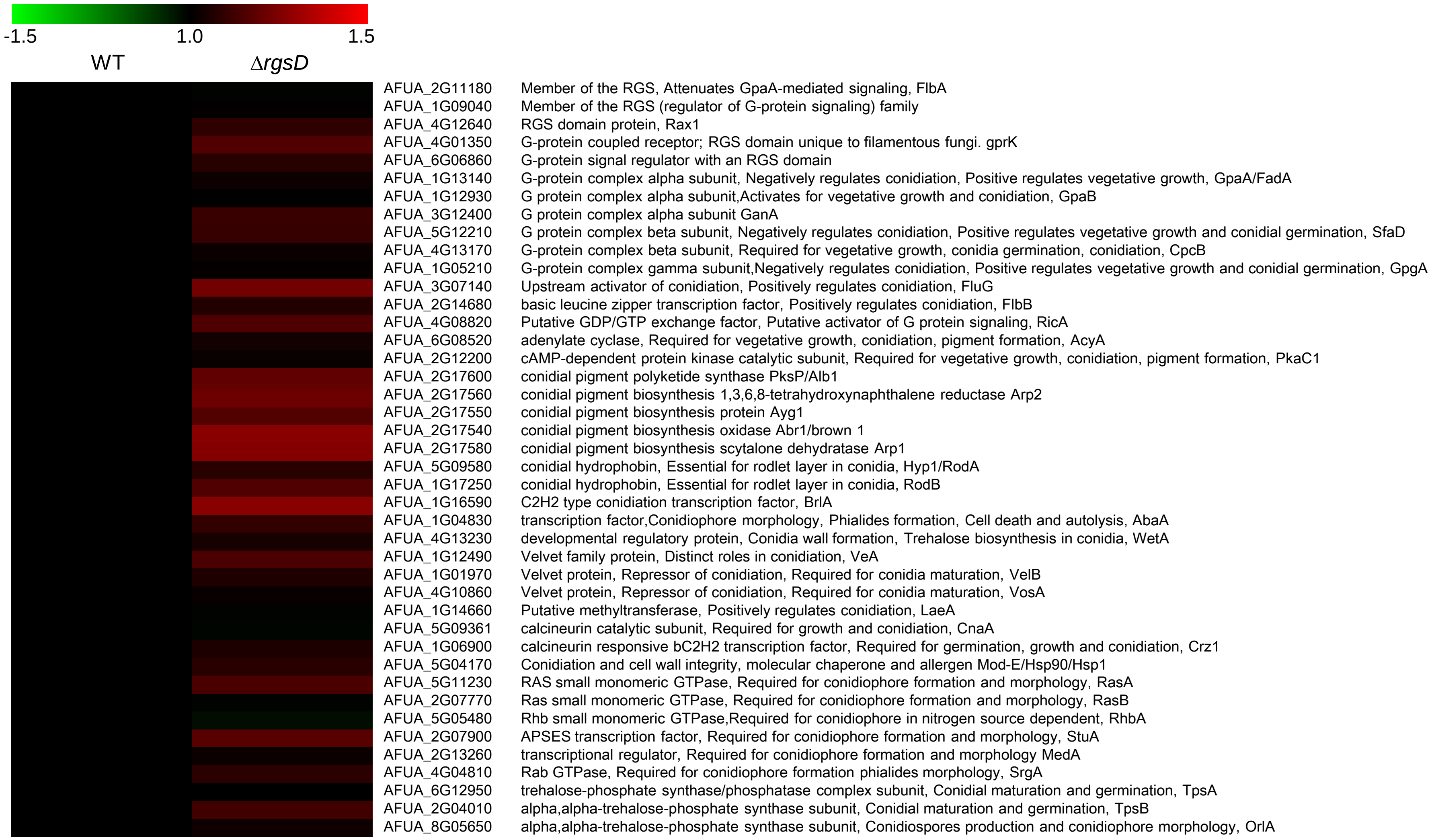

Figure S4. Verification of Δ*rgsD*. (A) Schematic illustration of the *rgsD* regions and restriction maps of WT, Δ*rgsD*, and complemented (C′) strains. (B) PCR amplicons for the three strains. Lane M, molecular weight marker. (C) The *Sac*I digestion pattern of individual amplicon. While the Δ*rgsD* amplicons are cut into two fragments, the WT and C′ amplicon remains uncut.

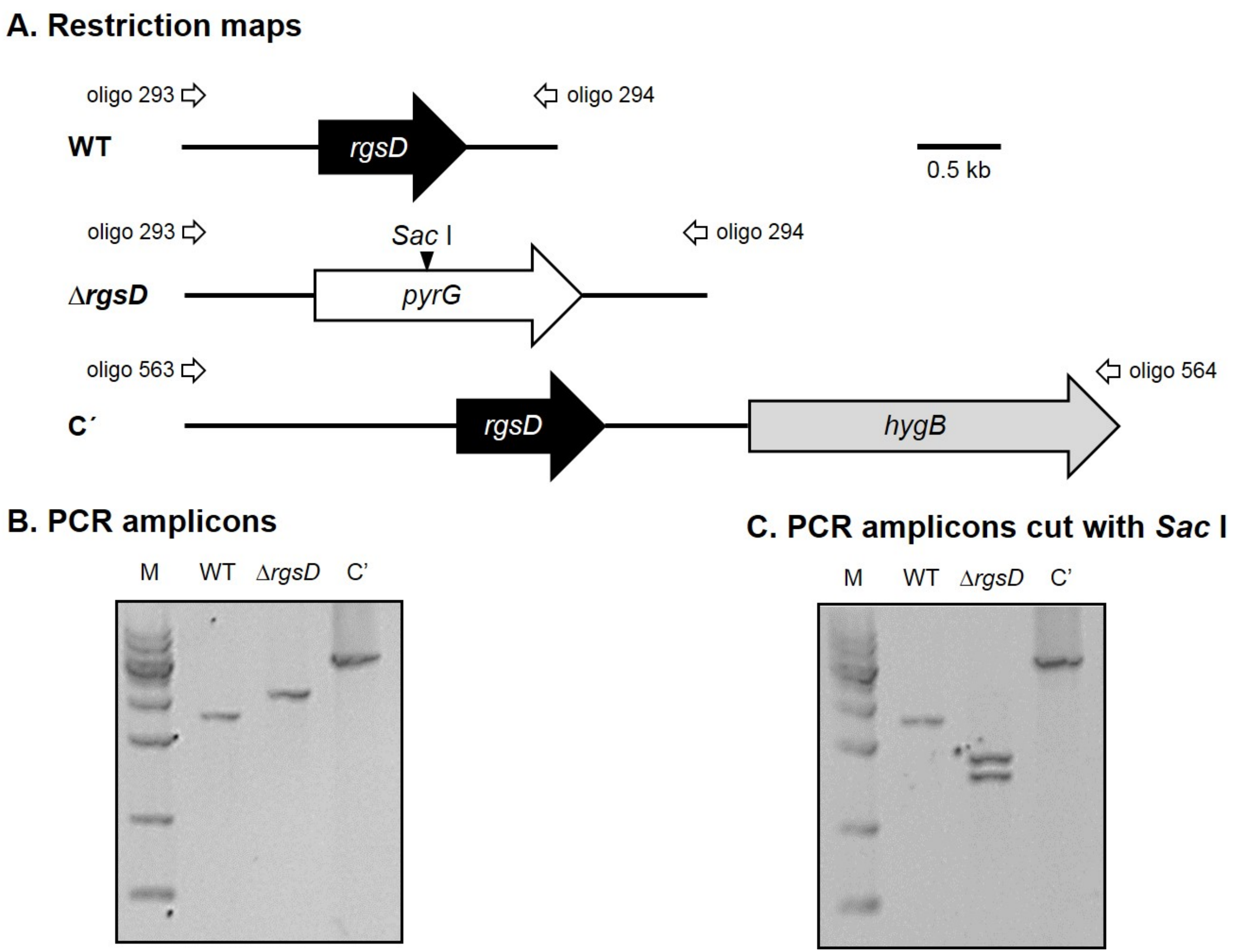

Supplementary Table S1. The oligonucleotides used to in this study.

Name	Sequence (5'→3')[a]	Purpose
oligo256	TTCCAAGCAGAGCTTGTCAC	5' *brlA* for qRT-PCR
oligo257	CCAGGTTCTTTGCACTTGAA	3' *brlA* for qRT-PCR
oligo271	AAATCCATCACATCCACCCT	5' *gliZ* for qRT-PCR
oligo272	GGTTGTTCATGGTCAGTTGC	3' *gliZ* for qRT-PCR
oligo303	GCTACCACTCTGCATCCTCA	5' *abaA* for qRT-PCR
oligo304	TACGAGCTCCAGCATGATTC	3' *abaA* for qRT-PCR
oligo305	ACGGCAGGAAGTTGTCTTCT'	5' *wetA* for qRT-PCR
oligo306	CTGTCAGCGACTTGTTGGAT	3' *wetA* for qRT-PCR
oligo307	ATGAACCAGCCAGGTACTCC	5' *vosA* for qRT-PCR
oligo308	GTTGGTCCCTTGTGGAAAGT	3' *vosA* for qRT-PCR
oligo346	CCATGTGTGTCGAGTCCTTC	5' *ef1* for qRT-PCR normalization
oligo347	GAACGTACAGCAACAGTCTGG	3' *ef1* for qRT-PCR normalization
oligo430	TTCCAAATGTGGCAAGTGAT	5' *acyA* for qRT-PCR
oligo431	GCAAACGTGGAATCAATACG	3' *acyA* for qRT-PCR
oligo432	GACCCAGCTAGGAACCACAT	5' *pksP* for qRT-PCR
oligo433	AGTGACACCAAGACCCTTCC	3' *pksP* for qRT-PCR

oligo434	CCACCACCTACAACAACAGC	5' *pkaC1* for qRT-PCR
oligo435	TGTGAAGACGCATGATGAGA	3' *pkaC1* for qRT-PCR
oligo605	GTATACGAGTAGCGCGGTGA	5' *rgsD* for qRT-PCR
oligo606	TCAGACCATTCCTTTGGACA	3' *rgsD* for qRT-PCR
oligo689	AAACCCCTGTGAATGCAGAC	5' *gliP* for qRT-PCR
oligo690	CCCCTTGAGATGAAAGGTGA	3' *gliP* for qRT-PCR
oligo691	CGATCTGTACCCCAACGAGT	5' *gliM* for qRT-PCR
oligo692	TTCTGGAACTTTGCCAGCTT	3' *gliM* for qRT-PCR
oligo693	ACTCCACCATCCAGTTCCAG	5' *gliT* for qRT-PCR
oligo694	TCCGAGTATCCCTCGATGTC	3' *gliT* for qRT-PCR
oligo726	CAGTGGAGGACCTTGCGTTA	5' *gpaB* for qRT-PCR
oligo727	GACTTTGGGACTCGTTGGTT	3' *gpaB* for qRT-PCR
oligo1024	AAGCCTCGCATCTATCACCA	5' *nsdD* for qRT-PCR
oligo1025	CGCTGTGGGTAGGTGATTTG	3' *nsdD* for qRT-PCR
oligo1026	ACCGTCTACAGTGCCAAGAA	5' *veA* for qRT-PCR
oligo1027	AGCAAAGGCCCTCTCATCAT	3' *veA* for qRT-PCR
oligo1050	GACTGCGAAAGTGGGATGAC	5' *arp1* for qRT-PCR
oligo1051	CCAATGACCTCGGTATCGGA	3' *arp1* for qRT-PCR

oligo1052	CTGGAGGACTTCAACGAGGT	5' *arp2* for qRT-PCR
oligo1053	CGGCAATTGAGGACATGAGG	3' *arp2* for qRT-PCR
oligo109	GCAATGTAAAGCTAACGTGCGTG	5' *AnpyrG* marker
oligo110	TGCCTTTAAGCTTCGGGTAGAG	3' *AnpyrG* marker
oligo289	ACTTCTGTCCTCGTGCGATT	5' flanking region of *rgsD*
oligo290	*TTTGTAGGCTTTGGGCTGTTCACAA*CAGCTATTTCAGCCCTTCGT	5' *rgsD* with *AnpyrG* tail
oligo291	ACCCATGAATAGCAGCATCC	3' flanking region of *rgsD*
oligo292	*CTGATCTACCCCTTGGAACGCAGCA*GGGATAAGATACCCAATGACGA	3' *rgsD* with *AnpyrG* tail
oligo293	TTCTTCACCATCTTCCAATGC	5' nested of *rgsD*
oligo294	GCAGACAAAATCGCCAGTCT	3' nested of *rgsD*
oligo559	TACGAATCGCGATACAACAATGTCTT	5' *rgsD* for complementation
oligo560	*CTTGACATGTGCAGCCGGTGGAG*CGATCTACCCATGAATAGCAGCATC	3' *rgsD* with *hygB* for complementation
oligo561	GATGCTGCTATTCATGGGTAGATCG*CTCCACCGGCTGCACATGTCAAG*	5' *hygB* with *rgsD* for complementation
oligo562	CCAGTCACTATGGCGTGCTGCTAG	3' nested of *hygB*
oligo563	GTCTGTCTCTGTGTCTTGGCCTGTT	5' of *hygB*

[a] Tail sequence is in red and italic.
